# Artificial Splitting of a Non‐Ribosomal Peptide Synthetase by Inserting Natural Docking Domains

**DOI:** 10.1002/anie.201915989

**Published:** 2020-05-27

**Authors:** Carsten Kegler, Helge B. Bode

**Affiliations:** ^1^ Molekulare Biotechnologie, Fachbereich Biowissenschaften Goethe Universität Frankfurt Max-von-Laue-Straße 9 60438 Frankfurt am Main Germany; ^2^ Buchmann Institute for Molecular Life Sciences (BMLS) Goethe-Universität Frankfurt 60438 Frankfurt Germany; ^3^ Senckenberg Gesellschaft für Naturforschung Senckenberganlage 25 60325 Frankfurt Germany

**Keywords:** docking domains, combinatorial biosynthesis, heterologous expression, non-ribosomal peptide synthetases, xefoampeptides

## Abstract

The interaction in multisubunit non‐ribosomal peptide synthetases (NRPSs) is mediated by docking domains that ensure the correct subunit‐to‐subunit interaction. We introduced natural docking domains into the three‐module xefoampeptide synthetase (XfpS) to create two to three artificial NRPS XfpS subunits. The enzymatic performance of the split biosynthesis was measured by absolute quantification of the products by HPLC‐ESI‐MS. The connecting role of the docking domains was probed by deleting integral parts of them. The peptide production data was compared to soluble protein amounts of the NRPS using SDS‐PAGE. Reduced peptide synthesis was not a result of reduced soluble NRPS concentration but a consequence of the deletion of vital docking domain parts. Splitting the xefoampeptide biosynthesis polypeptide by introducing docking domains was feasible and resulted in higher amounts of product in one of the two tested split‐module cases compared to the full‐length wild‐type enzyme.

## Graphical abstract

**A breed apart**: The non‐ribosomal peptide synthetase (NRPS) XfpS, which produces the peptides xefoampeptides A and B, can be artificially split without loss of biosynthetic activity by introducing docking domains into XfpS. In fact, in this case, the production of both peptides from the split system increased compared to product levels from the full‐length XfpS. Splitting XfpS into three subunits also led to a functional NRPS system.

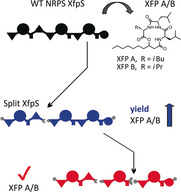

Several clinically used drugs including antibiotics and anticancer and immunosuppressive drugs are generated by non‐ribosomal peptide synthetases (NRPSs). While we understand very well how these megaenzymes work biochemically (Figure 1), they are often difficult to manipulate due to their size. In NRPS systems that consist of more than one polypeptide or subunit, these subunits must be specifically and non‐covalently linked for a successful biosynthetic outcome. In classical NRPSs, different subunits selectively interact following the collinearity rule, thereby giving rise to the synthesis of peptides with defined sequences. The specific interprotein interactions are mediated by N‐ and C‐terminally located matching pairs of short docking domains (DDs)^1^, ^2^ or communication‐mediating domains (COM).^3^, ^4^ Stachelhaus and co‐workers demonstrated that for the NRPS systems synthesizing tyrocidine, gramicidin, and surfactin, COM swapping led to enzyme crosstalk between biosynthetic systems, thereby promoting the combinatorial biosynthesis of different peptides.^4^, ^5^, ^6^ While the condensation (C)‐domain side of the COM domain is integrated into the C‐domain surface itself,^7^ DD domains connecting epimerization (E) to C, thiolation (T) to C, or T to cyclisation (Cyc) domains have been described.^8^, ^9^, ^10^

**Figure 1:**
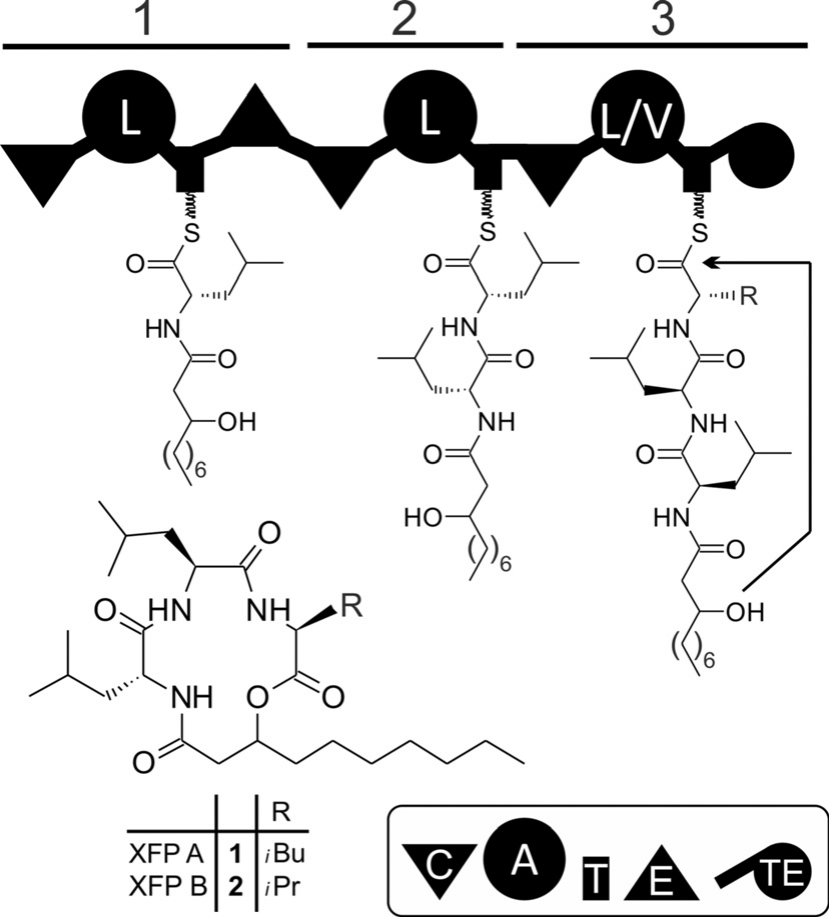
Biosynthesis of xefoampeptides (XFP) A (**1**) and B (**2**), highlighting the NRPS mechanism and domain organization. A=adenylation domain; T=thiolation domain; C=condensation domain, E=epimerization domain; TE=thioesterase domain.

In contrast to COM domains, the structure of an elucidated ^N^DD from a hybrid system of a polyketide synthase and NRPS was shown to be appended to a Cyc domain.^9^ All ^C^DD‐^N^DD interactions for a complete NRPS system including the first structure of a ^C^DD‐^N^DD complex have been defined for the rhabdopeptide‐synthesizing NRPS, detailing the key amino acids residues of the DD interaction surface.^2^ A similar DD pair fold [β‐hairpin docking (βHD) as ^N^DD of a C‐domain interacting with short linear motifs (SLiMs) as ^C^DD of a T‐domain] have been observed in the enacyloxin biosynthesis,^10^ while a new NRPS DD fold only based on α‐helical structures has been observed for the PaxABC NRPS system.^11^ Understanding DD interfaces and using them to split up multimodular NRPS biosynthesis would be highly desirable in the quest to redesign and engineer large NRPSs.^12^, ^13^ The NRPS multienzyme machinery often consist of huge single subunits containing multiple modules, with each one of them comprising 120 kDa so that, for example, the single‐protein six‐module NRPS AmbS^14^ is in fact a 739 kDa protein encoded on a gene of 20 kb length. Creating smaller NRPS subunits could facilitate overproduction of NRPSs for protein purification and in vitro studies by splitting the metabolomic burden for the individual cell. The feasibility of subunit splitting by introduction of DDs has been demonstrated for the pikromyin polyketide synthase with little loss of productivity.^15^

Here, we show the applicability of splitting multimodule NRPSs by natural DD pairs. As a model, we used the xefoampeptide (XFP)‐producing NRPS XfpS from *X. bovienii*. It comprises three modules that produce the main products XFP A (**1**) and XFP B (**2**), which are very stable in aqueous solutions and show weak bioactivity against *Trypanosoma cruzi*, and XFP production was known to be highly reproducible in *E. coli*. Even more importantly, XfpS, being a relatively small NRPS, encompasses the two typical module domain interfaces connected by DDs in *Xenorhabdus*, namely, the epimerization domain (E1) to the condensation domain (C2) and the thiolation domain (T2) to condensation domain (C3) interface (Figure 1).

The *xfpS* gene encoding the XFP‐producing NRPS XfpS was amplified from genomic DNA of *X. bovienii*, cloned into an expression vector under control of an l‐arabinose‐inducible P_BAD_ promoter, and expressed in *E. coli*. Best production of XFP was achieved at 22 °C, which was used for all subsequent experiments also for other *xfpS* variants (see below). Cultures without the transcriptional activator l‐arabinose were devoid of any **1** and **2**. Since we originally wanted to track the amount of soluble XfpS or its derivatives by western blotting, all XfpS variants were N‐ and C‐terminally tagged with a Strep‐Tag II.^16^, ^17^ Upon comparing the tagged to untagged XfpS using a quantitative data set of XFP being produced (Figure 2 a and b), a significant 2.5‐fold increase of XFP biosynthesis was noticed as an effect of a N‐ and C‐terminal Strep‐Tag II. The tag itself is an artificially developed affinity tag described as biologically inert and proteolytically stable.^16^, ^17^ The cause for this increase of productivity might be a changed half‐life of XfpS, effects on the translational initiation, or effects on the mRNA transcript, but this was not analyzed in detail in this work.

**Figure 2:**
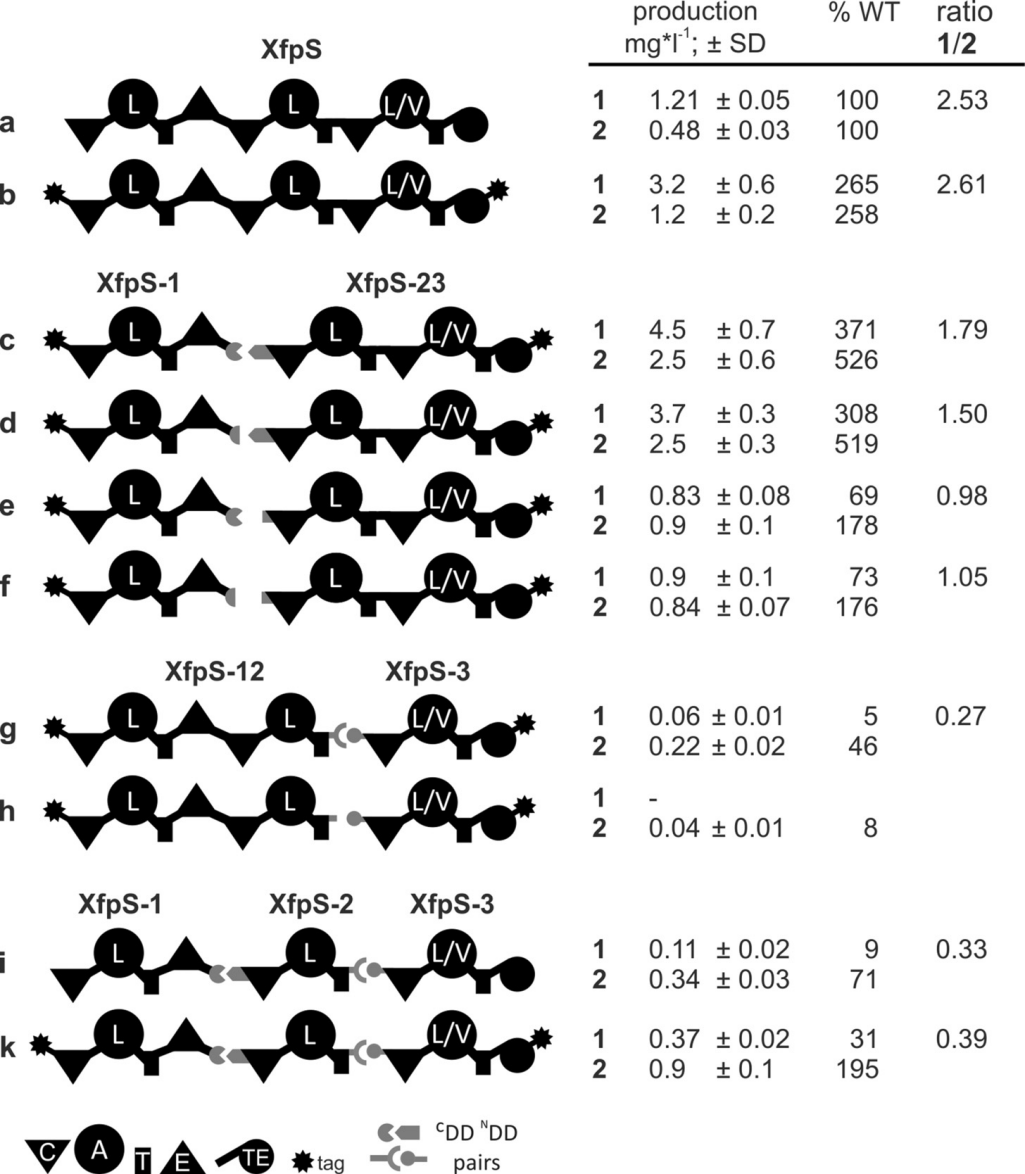
Schematic overview of XfpS subunit arrangements and XFP production (±SD).

In the attempt to split XfpS into module pairs and stand‐alone modules, docking‐domain (DD) pairs known to produce peptides were selected (for more details see the methods section in the Supporting Information). For the E‐ to C‐domain split, a DD pair from the *X. bovienii* biosynthetic gene cluster *txlAB* coding for the taxlllaid‐producing NRPS was selected,^2^, ^9^, ^18^ which represents the only DD type in *Xenorhabdus* connecting E to C domains. The *txlA* ultimate 53 codons were fused following *xfpS* codon T1442, thereby introducing the *txlA* ^C^DD region and stop codon (for details see Figure S3 and the Supporting Information). The first 91 *txlB* codons were cloned in frame prior to *xfpS* codon Y1476, introducing the translational start codon, the translational start site, and the ^N^DD coding region. The DDs for introduction into the T2‐C3 interface was drawn from the *X. bovienii* gene cluster *paxABC* homologue described for *X. nematophila* HGB081.^19^ This DD pair is found to exclusively link T to C domains representing a new DD class.^11^ The *paxB*‐encoded ^C^DD comprising 37 codons was integrated following *xfpS* L2504 codon insertion into the ^C^DD region and a translational stop. The first 48 *paxC* codons were cloned in front of the *xfpS* codon F2526, including the start ATG and a Shine–Dalgarno region (SD; Figure S3). The DD introduction resulted in two XfpS versions of XfpS non‐covalently connected by DDs, namely XfpS‐1 (first module of XtpS) to XfpS‐23 (XtpS module 2 and 3) and XfpS‐12 (XtpS module 1 and 2) to XfpS‐3 (XtpS module 3; Figure 2 c and g). The imported DD pair coding region from *txlAB* and *paxABC* transferred the capacity to translationally initiate the second NRPS multienzyme in each series most likely by translational coupling.

The two polypeptide NRPS versions connected by DDs were both biosynthetically active. Connecting XfpS module two to XfpS three (via DDs connecting XtpS‐12 to XtpS‐3) by T/C domain‐connecting DDs (Figure 2 g) produced 5 % and 46 % of **1** and **2**, respectively, when compared to the original wildtype NRPS (Figure 2 a). The integration of DDs between modules one and two in Figure 2 c (XfpS‐1 to XfpS‐23) increased the production of **1** and **2** significantly compared to the wildtype NRPS (Figure 2 a).

In order to confirm that the inserted DD pairs are indeed required for the observed peptide production, we generated a set of XfpS variants showing DD deletions that do not interrupt the translational coupling^20^ of the gene cluster (Figure S4). Since the start‐ATG following codons plays a crucial role in the translational initiation process,^21^ the ^N^DD deletion left the first ten codons in place with the objective to maintain the same molar ratio of XfpS subunits to each other.

Three separate deletions were tested for the E/C DD‐pair NRPS (Figure 2 c). Deletion of 31 ^C^DD codons of the 53 amino acid DD (Figure 2 d), deletion of 61 codons of the 91 amino acid ^N^DD (Figure 2 e; for details see Figure S3), or a combination of both deletions were carried out (Figure 2 f). While deletion of the ^C^DD alone (Figure 2 d) was indistinguishable from the ancestor bearing full‐length DD (Figure 2 c), deletion of the ^N^DD alone and of both DDs resulted in a significant drop in production of **1** and **2** compared to the ancestor bearing the full‐length DD (Figure 2 c). The above described deletion strategy left the seven final ^C^DD amino acids untouched, which confer interaction with the β‐hairpin docking (βHD) ^N^DD^2^, ^10^ while substantially shortening the connection of the E domain to the ^C^DD. The ^N^DD deletion (Figure 2 e,f) erases the entire β‐hairpin part of the ^N^DD to which the ^C^DD binds. For the DD pair connecting the T2 domain to the C3 domain non‐covalently (Figure 2 g), 30 of 37 ^C^DD codons were deleted (Figure 2 h; Figure S3C and S4), resulting in a severe drop in XFP productivity.

The change in XFP synthesis in all of the presented artificial XfpS gene clusters could be a consequence of the capacity of DDs to connect the modules in the biosynthetic context or it could be a measure of the functional soluble protein in the cell. The DD deletions in particular could potentially result in reduced XfpS concentrations in the cell. Since western blotting identification of the NRPS parts had failed, we attempted to visualise soluble XfpS proteins using SDS‐PAGE. As can be seen in Figure 3, the full length three‐module XfpS and all two‐module XfpS subunits were clearly detectable [Figure 3; XfpS‐123 (the full length XtpS), XfpS‐12 and XfpS‐23], identified by their size and in contrast to the non‐induced control. The single‐module XpfS‐1 could not be detected due to other overlapping proteins but the production rate of the delta‐^C^DD (Figure 2 d) did not cause any change in the coupled translation of XfpS‐23 judging from the intensities of soluble protein bands while the XFP production was indistinguishable (compare Figure 2 c and d). By contrast, the drop in XFP production detected in Figure 2 e and f does not correlate with a drop in the protein amount of XfpS‐23.

**Figure 3:**
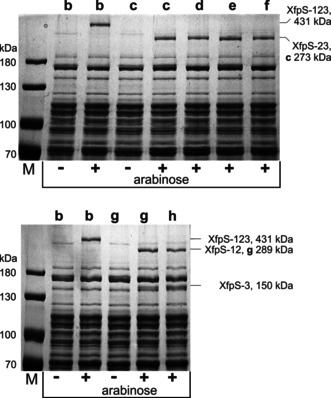
Coomassie‐blue‐stained SDS‐PAGE of soluble protein of *E. coli* heterologously expressing XfpS variants at 22 °C. Letters **b**–**h** correlate to designations in Figure 2. The assignment of XfpS subunits is indicated to the right of the gels. The exact molecular masses of all XfpS proteins are listed in the method section and a protein marker is shown on the left. Arabinose functions as transcriptional activator of *P_BAD_* in front of *xfpS*.

It was possible to detect XfpS‐3 (Figure 2 g) as well as its DD‐deletion variant (Figure 2 h) in relation to the non‐induced *E. coli* strain (Figure S5). Amounts of XfpS‐3 in both experiments are similar and in the same molar range as XfpS‐12 (Figure 3), thus suggesting that the ^C^DD deletion does not affect the amount translationally coupled XfpS‐3. The assessment of protein amounts using SDS‐PAGE is certainly a semiquantitative approach, however, the drop in XFP production (Figure 2 h) is undoubtedly not a result of a drop in XfpS subunits but can be attributed to the impairment of DD functioning as a result of the deletion within the ^C^DD.

Finally, the two DD pairs used to create different two‐subunit XfpS variants were combined, leading to an NRPS comprising three individual stand‐alone modules (XfpS‐1, ‐2, and ‐3) transcriptionally regulated by one *P_BAD_* promoter (Figure 2 i). This artificial three‐gene cluster undoubtedly led to a biosynthetically active multienzyme complex. Since construct **i** was untagged, it was necessary to compare it to the tagged version in order to validate the product increase observed from **a** to **b**. The tagged version of the three individual stand‐alone XfpS modules (Figure 2 k) produced 2.7 times the amount of **1** and 3.3 times of **2** compared to construct **i** and outperforms **g** but falls short of reaching the production levels of the best producer **c** and **d**. Since all of the XfpS‐2 to XfpS‐3 constructs resulted in lower product yield compared to **c**, other DDs for the T2 to C3 split were tested. DDs from two xenoamicin‐producing NRPSs^22^ were introduced into construct **k**, resulting in **m** and **n**, which were not able to surpass production from **k** and the skewed **1**/**2** product ratio remained (see Figure S7).

Reflecting on the results of the T‐to‐C splits, we examined the recently published LgrA C‐domain/T‐domain interaction structural data.^23^ In the elongation cycle, the T domain interacts with most of its surface with the C domain but the outside‐facing part of the fourth α‐helix is solvent‐exposed. Hence, it could be that ^N^DD interaction with the ^C^DD could extent into structural parts of the T domain. Thus, construct **n** shifted the PaxA/B DD fusion point into α‐helix 4 and construct **o** exchanged the entire fourth T2‐α‐helix of XfpS (see Figure S7). No production is detectable with **n** but production is restored to above 50 % levels relative to **k** for **o**, thus strongly suggesting a role for the T domain in the DD interaction. Furthermore, the product ratio in **o** shifts slightly back towards that of XfpS (**a** and **b**). The product ratio skew in all DD cases linking XfpS‐2 to XfpS‐3 constructs is interpreted as a measure for some sort of impaired movement of domains in the elongation cycle of the NRPS since all constructs dock and produce well above the DD‐deletion control **h**. Structural information about how the ^N^DD interacts with the T domain will likely to be the way forward to solve this production impediment.

In this work the artificial splitting of a single multienzyme NRPS was achieved through the introduction of DDs whilst maintaining the biosynthetic activity and even increasing it in some cases. The quantification of peptide product in tandem with monitoring the in vivo soluble NRPS protein concentration is was valuable path to gain insight into decoding the reasons for NRPS biosynthetic activity. The chosen method to import the translational coupling capacity from a given gene cluster turned out to be successful. The DD‐deletion strategy in which a crucial translational stop was imported to the translational start junction of the translational initiation process was key to preserving the same number of NRPS subunits. The same numbers of subunits in turn was important for the analysis so that observed effects could be attributed only to the DDs and deletions within the DDs but not to different molar numbers of subunits. Deletion of large parts of the DDs did not terminate NRP biosynthesis entirely, in contrast to the total loss of enzymatic activity in the case of COM domains connecting TycAB.^4^

The DD‐deletion controls shed light on their role in coupling NRPS modules. They seem to be more important when connecting T with C domains and less important for the connection of E and C domains. Deletion of large DD parts never reduced product synthesis below 8 %. Deletion of 6 DD amino acids at their cognate cluster position in the COM‐TycAB case^4^ completely abolished the biosynthesis. Moreover it has been shown that the change of a single, crucial amino acid of a DD pair reduces the product yield below 1 %^24^ and can increase the DD *K*_d_ value by more than twelvefold.^2^ In contrast to the above, the relatively high XfpS production rates in **e** and **f,** in which the ^C^DD‐^N^DD interaction parts^2^ were deleted, can be attributed to a capacity of E and C domains to associate without DDs, probably due to the large interaction surface of both domains, while T‐to‐C domain splits seem to be more reliant on DDs due to the smaller protein—protein interaction interface.

The presented data suggest that the artificial DD splitting approach between E‐ and C‐domain modules can be incorporated into the expanding efforts of de novo design of NRPSs.^6^, ^12^, ^13^, ^25^ Being able to create smaller NRPS genes of a cluster without losing enzymatic activity, increases engineering flexibility, for example, by enabling the coding of smaller de novo engineered subunits^12^, ^13^, ^26^ on different vectors, thereby allowing any de novo NRPS subunit on one vector to be combined with any de novo change on the other. It has not slipped our attention that adding N‐ and C‐terminal protein tags to the NRPS led to a 2.5‐fold increase in XFP production and this approach will be applied and further explored in the future.

## Conflict of interest

The authors declare no conflict of interest.

## Supporting information

As a service to our authors and readers, this journal provides supporting information supplied by the authors. Such materials are peer reviewed and may be re‐organized for online delivery, but are not copy‐edited or typeset. Technical support issues arising from supporting information (other than missing files) should be addressed to the authors.

Supplementary
