# Supplementary material for: Artificial Splitting of a Non‐Ribosomal Peptide Synthetase by Inserting Natural Docking Domains
Source: Angew Chem Int Ed Engl. 2020 May 27;59(32):13463–7. doi: 10.1002/anie.201915989 (PMC7496407; doi:10.1002/anie.201915989)
Supplement: Supplementary file 1 — Supplementary [file ANIE-59-13463-s001.pdf]

Angewandte Chemie
Eine Zeitschrift der Gesellschaft Deutscher Chemiker

Supporting Information

Artificial Splitting of a Non-Ribosomal Peptide Synthetase by Inserting Natural Docking Domains

*Carsten Kegler and Helge B. Bode**

anie_201915989_sm_miscellaneous_information.pdf

Supporting Information

Table of content

Methods used in this work.

Introduction of docking domains by fusion into *xfpS.*

Heterologous production in *E. coli* DH10B::*mtaA*.

Methanolic extraction of compounds in cultures for HPLC/MS analysis.

Bioactivity of XFP A/B

Quantification of XFP A/B production in E. coli DH10B::*mtaA*.

UPLC-ESI-MS analyses.

Heterologous protein expression and extraction for SDS-PAGE analysis.

Documentation of SDS-PAGE images and digital processing.

Cloning of plasmids in this work.

Figure S1. UPLC-ESI-MS, analysis of XFP A/B (**1**/**2**) from *X. bovienii* heterologously produced in *E. coli*.

Figure S2. UPLC-ESI-MS, MS^2 analysis of XFP A/B (**1**/**2**) from *X. bovienii* heterologously produced in *E. coli*.

Figure S3. Alignment of DDs with ΔDD counterparts.

Figure S4. DD-deletion and translational coupling cloning approach.

Figure S5. SDS-PAGE of soluble *E. coli* DH10B expressing XfpS constructs.

Table S1. Primer used in this work.

Table S2. Plasmids used in this work.

Table S3. Bacterial strains used in this work.

References

Methods used in this work

Introduction of docking domains by fusion into *xfpS.*

The rationale behind the suitability of the DD was guided by the idea to select DD from the related genera *Xenorhabdus* and *Photorhabdus*. In the case the E to C domain module interface, all accessible genomes of *Xenorhabdus* and *Photorhabdus* species for NRPS gene clusters with DDs were analyzed. Only two DD types could be found of which one is likely to be the COM-domain kind [1]. This option was excluded as the COM-domain relies on a 'hand-motif' within the C domain itself which is not part of the XfpS C2 to be connected. The remaining E to C domain connecting DDs found in the genome search fell into the published DD type from *Xenorhabdus stockiae* KJ12.1[2] (see supplementary Fig. 2 therein) structurally related to the formerly published TubC-^{N}DD NRPS[3]**,** EpoB ^{N}DD[4] and β-hairpin docking (βHD) type[5] all of which consist of the same αββαα topology.

The strategy to choose the exact fusion point (see below) depended on a ^{C}DD connected to an E domain thereby preventing the use of ^{C}DDs attached to T domains.The DD pair from the *X. bovienii txlAB* gene cluster (*txlA* locus tag: XBJ1_0775, protein id: CBJ79916.1; *txlB* locus tag: XBJ1_0774, protein id: CBJ79915.1) was selected as DD pair to connect the XfpS module interface E1 to C2.The exact fusion site position of XfpS for the module E1-domain to TxlA-^{C}DD was based on the crystal structure of TycA[6]; PDB 2XHG). The TycA structure ends at its C-terminal site with a long α-helix. The identification of this α-helix in the XfpS E-domain was guided by XfpS to TycA E1-domain alignment and, more importantly, by using secondary structure prediction[7]. XfpS was left untouched up to T1442 followed by an in frame fusion of the 53 ^{C}DD *txlA* codons for G4634 to V4686. The fusion site of the ^{N}DD of TxlB to the XfpS C2-domain was based sequence alignments to the SrfA-C crystal structure[8], the Cyc-domain structure of EpoB[4] to the XfpS C2-domain. The TxlB ^{N}DD (M1-I91, 91 aa) was fused in front of Y1476 of XfpS. The deletion of the *txlA* coding ^{C}DD part leading to XfpS **d** and **f** comprised 31 of 53 codons leaving the C-terminal 7 codons in place (see Fig. S3). The deletion of the *txlB* coding ^{N}DD part (delta R12 to N61) leading to XfpS **e** and **f** comprised 49 of 91 codons leaving the first 11 codons in place. The ^{N}DD truncation deleted the described ^{C}DD to ^{N}DD interaction surface[2].

DDs connecting T to C domains are diverse in the genera *Xenorhabdus* and *Photorhabdus* and, with the exception DD connecting NRPS in the rhabdopeptide/xenortide synthesis[9], have not been structurally elucidated. For the above mentioned published rhabdopeptide DD group, it has been proven that the DDs allow a high degree of promiscuity in their NRPS connection capability and, moreover, are of the same class used to connect E to C domains. We focused our DD choice to the ones from the Xenoamicin NRPS[10] and the PAX producing NRPS[11] as (i) the synthesis is known, (ii) the domain flanking DDs operate to connect T to C domains specifically, (iii) the gene clusters are widespread in the genera, thereby guiding the definition of exact DD boarders, (iv) DDs have been successfully used to reprogram promiscuous rhabdopeptides/xenortides to specific ones[12], (v) they are a different DD type than the αββαα topology fold group, and (vi) their structure was recently solved[13]. In Fig. S6 the alignment of the PaxABC NRPS T-to-^{C}DD and the ^{N}DD-to-C domain region with the XfpS relevant regions is shown.

The DD pair PaxBC originating from *X. bovienii paxABC* gene cluster[11] (*paxB* locus tag: XBJ1_2152, PaxB protein id: CBJ81278.1; *paxC* locus tag: XBJ1_2151, PaxC protein id: CBJ81277.1) was selected as DD pair to connect the XfpS module interface the T2 to C3. To select a suitable ^{C}DD integration site the XfpS-T2 domain was aligned with the PaxB domain T4 and the SrfA-C T-domain[8]. The end of the C-terminal α-helix of the SrfA-C T-domain was chosen as fusion spot of XfpS-T2 to the ^{C}DD of PaxB. As a result, XfpS was left untouched up to L2504 being fused to the ^{C}DD 37 codons for L3285 to Q3321 of PaxB. The PaxC ^{N}DD coding region comprised 48 codons (M1-S48) and was integrated into XfpS in front of F2526. The ^{C}DD deletion in XfpS **g** comprised 30 of the 37 codons of the PaxB leaving the ultimate C-terminal 5 codons in place (see Fig. S3). Construct **g** was further modified by integrating parts of the T2 domain α-helix4 from PaxA into the XfpS T2 domain (Fig. S7) resulting in construct **n**. Incorporation of the entire PaxB α-helix4 into the XfpS T2 domain led to construct **o** (Fig. S7). The DD from XabAB and the XacAB NRPS are exclusively found to link T to C domains. The XabAB DD was taken from *X. doucetiae* (*xabA*, XDD1_2281, UniProt A0A068QVS6; *xabB* XDD1_2282, UniProt A0A068QTK0) and integrated into XfpS between T2 and C3 identical to the PaxAB DD resulting in XfpS **i**. The XacAB DD was taken from *X. doucetiae* (*xabA*, XDD1_2286, UniProt A0A068QVT0; *xabB* XDD1_2287, UniProt A0A068QTK5) and integrated into XfpS between the T2 and C3 domain identical to the PaxAB DD, resulting in XfpS **m**.

The import of DDs into XfpS implied that a second, or second and third, translational initiation event had to take place so that all three XfpS modules can be translated. This was achieved by simply importing the TxlAB, PaxBC, XabAB and XacAB translational termination coupled to the translational initiation context into *xfpS*. The above described DD deletion constructs were carefully designed to preserve the translational coupling capacity (see Fig. S4).

Heterologous production in *E. coli* DH10B::*mtaA*. Heterologous production of **1** and **2** started with a 20 ml LB (kanamycin 50 µg/l, chloramphenicol 20 µg/l) overnight culture in a 100 ml Erlenmeyer flask cultivated at 30°C (shaking at 200 180 rpm). The following day 200 µl of the overnight culture were used for inoculation of 20 ml LB containing kanamycin (50 µg/l), chloramphenicol (20 µg/l), 1 % (v/v) Amberlite® XAD-16 resin (Sigma-Aldrich) and 0.2% L-arabinose as transcriptional activator of P_{BAD}[14]. Production cultures were then cultivated at 200 rpm at 30°C for 24 h or at 26°C for 48 h or at 22°C for 72 h. The heterologous production for the absolute quantification data shown in figure 1 was solely carried out at 22°C. *E. coli* cultures and XAD-16 beads were finally harvested by centrifugation (4°C, 4500 g, 15 minutes) after which the emerged supernatant was discharged.

Methanolic extraction of compounds in cultures for HPLC/MS analysis. Pelleted cells and XAD-16 from production cultures were incubated with 20 ml methanol for 20 minutes at constant agitation. The methanol was then separated from cells and XAD-16 beads by passaging it through a fluted filter. The filtered methanol extract was centrifuged at 17000 g at room temperature for 15 minutes. The cleared methanol extract supernatant was diluted appropriately for HPLC-MS quantification measurements.

Bioactivity of XFP A/B. XFP A (**1**) was tested against trypomastigote forms of *Trypanosoma cruzi* Tulahuen C4 (IC_{50} of 55.1 µg/mL) according to the previously published protocol[15]. *T. cruzi* is the protozoa causing the Chagas disease also known as American trypanosomiasis[16].

Quantification of XFP A/B production in *E. coli* DH10B::*mtaA*. Compounds **1** and **2** were quantified based on a calibration curve from HPLC/MS triplicate

measurements. As standard isolated **2** (for quantification of **1** and **2**) was taken. To calculate the absolute production titres in *E. coli* DH10B::*mtaA* the pure isolated compound was diluted to the following concentrations as triplicates: 10, 2, 0.5, 0.1, 0.025 µg/ml. The peak area for the compound at the given concentration was calculated using DataAnalysis Version 4.3 (Build 110.102.1532) program. The compound peak area to MS-signal ratio in the measured range displayed a linear relationship. The linear regression equation derived from the standard curve for 2 is y = 1.511 e-9*x + 0.03306 (x equals peak area, y equals concentration of compound). For quantification of XFP A/B (**1**/**2**) at least four independent cultures were cultivated, extracted and quantified. The standard deviations of the calculated values were deduced for **1** and **2** for each examined construct.

UPLC-ESI-MS analyses. UPLC-ESI-MS analysis was performed on Ultimate 3000 LC system (Dionex) coupled to the AmaZon X electrospray ionization mass spectrometer (Bruker). Separation was achieved by a C18 ACQUITY UPLC™ BEH column (130Å, 1.7 µm particle size, 2.1 mm x 50 mm Waters). Acetonitrile/water containing 0.1% formic acid was used as mobile phase at a flow rate of 0.4 ml/min. The gradient started continuously with 5% acetonitrile for 2 minutes followed by a linear gradient ranging from 5 to 95% of acetonitrile over 16 minutes. The chromatography ended with an equilibration phase of 2 minutes at 95% acetonitrile. 10 µl of crude methanol extract was diluted in 90 µl methanol prior to analysis. The injection volume of a single UPLC-ESI-MS analysis was 5 µl.

Heterologous protein expression and extraction for SDS-PAGE analysis. Heterologous production of XfpS in *E. coli* DH10B::*mtaA* was identical to the above described production culture with the exception, that no XAD-16 was added. All protein extract cultures for SDS-PAGE analysis were executed at 22°C and cells from a 20 ml cultures were harvested 24 h post inoculation by centrifugation at 4°C at 4500 g for 20 minutes. The supernatant was discharged and the cell pellet resuspended in a tenth of the culture volume. In order to obtain similar protein concentrations in the protein extracts the volume for protein lysis was referenced to an OD_{600} of 2.5. Cells were lysed in a buffer containing 100 mM HEPES pH 7.6 at 25°C, 0.1% Triton-X-100, 250 mM NaCl, lysozyme, cOmplete™ protease inhibitor cocktail (Roche Diagnostics), 1 mM EDTA, 1 mM DTT. Resuspended cells were incubated at 4°C for 10 minutes followed by ultrasonification being cooled with ice water constantly. The soluble protein

extract was cleared by centrifugation at 4°C, 21000 g for 30 minutes. Two volumes of soluble protein containing supernatant were immediately mixed with one volume SDS-PAGE loading buffer. Proteins were size separated employing discontinuous Tris-glycine SDS-PAGE system consisting of a pH 6.8, 5% stacking gel and pH 8.8, 8% resolving gel (37.5:1 acrylamide to bisacrylamide). The molecular masses for the XfpS derivatives in this work are:

a, WT XfpS, 428.6 kDa;

b tagged XfpS, 431.5 kDa,

c, XfpS-1, 171.3 kDa; **c**, XfpS-23, 273.3 kDa;

d XfpS-1, 167.8 kDa; **d**, XfpS-23, 273.3 kDa;

e, XfpS-1, 171.3 kDa; **e**, XfpS-23, 266.2 kDa;

f, XfpS-1, 167.8 kDa; **f**, XfpS-23, 266.2 kDa;

g, XfpS-12, 289,7 kDa; **g**, XfpS-3, 149.6 kDa;

h, XfpS-12, 286,1 kDa; **h**, XfpS-3, 149.6 kDa;

i, XfpS-1, 169.8 kDa; **i**, XfpS-2, 131.5 kDa; **i**, XfpS-3, 148.2 kDa.

Documentation of SDS-PAGE images and digital processing. The SDS-PAGE shown in Fig. 3 and Fig. S5 was done in the following consistent processing steps. The original pictures were taken with a Nikon D-800/Micro-Nikkor 60mm F/2,8. The Coomassie stained SDS-PAGE were placed on a LED screen. The pictures were stored as raw-files in the Nikon Electronic Format (NEF). The original NEF-files are attached as documentation for the state of the unprocessed digital read-out (see files DSC_1320, DSC_6678.NEF and DSC_6680.NEF). All the following digital processing steps were applied equally to all of the picture as displayed in the Fig. 3 and Fig. S5. The first digital processing steps were done using Adobe Lightroom. The RAW interpretation of the pictures were then exported as .psd file. Further tailoring of the digital electrophoreses documentation (tilting, cutting, conversion to black and white, contrast adjustments) was performed using Adobe Photoshop CS4 extended version 11.0. The mask tool and merging of mask application has not used at any time in the process. The final interpretation of the pictures was exported in the Tagged Image File Format (.tif) which served as the format to import the picture into CorelDRAW 2018 (Version 20.1.0.708) in which the final figure was assembled.

Cloning of plasmids in this work. All the heterologous expression vectors described in this work were derived from the Novagen (Merck-Millipore) vector DUET series in a couple of steps. In a first step pCOLADuet was amplified via PCR using the primer pair pCOLA-NheI_for and pCOLA-NheI_rev. The PCR product was religated leading to the deletion of the *Nhe*I restriction site in the resulting vector **pCOLA-NheI**. pCOLA-NheI then was subjected to a restriction endonuclease digest using *Pst*I and *Nde*I. The linearized open vector was used to ligate the annealed oligos tacI_up/tacI_dw into the *Nde*I/*Pst*I sides leading to the vector **pCOLA_tacI** and thereby replacing the first T7 promoter against a *tacI* promoter. pCOLA_tacI was again linearized employing the restriction enzymes *Eco*RI and *Nco*I in order to ligate the annealed oligos tacI_NcoI_up and tacI_NcoI_dw and by doing so exchanging the second T7 promoter with the tacI promoter. The resulting vector was designated as **pCOLA_tacI/I**.
The vector pCOLA_ara/tacI[17]was used to introduce *araE* from Bl21 (DE3) into pCOLA_ara/tacI transcriptionally driven by *tacI*. As template Bl21(DE3) gDNA was adopted for the PCR in conjunction with the primer pair araE_Gib_for/araE_Gib_rev amplifying the insert fragment for a Gibson cloning reaction[18]. As vector part for this cloning step *Nde*I/*Xho*I linearized pCOLA_ara/tacI was fused with the *araE* containing DNA PCR fragment bringing **pCK_0412** into being.
A complementary set of DUET-derived vectors carrying the p15A ori of replication and the chloramphenicol acetyltransferase gene as antibiotic resistance marker was cloned. The aim of this gene manipulation effort was to insert the same *araC*-P_{BAD}-*tacI*-(*mtaA* or *araE*) cassette to a compatible but different origin-of- replication-antibiotic-resistance-marker combination so that two plasmids can be propagated within one *E. coli* cell. The vector pACYCDuet was taken as template in a PCR to delete the *Nhe*I site with primer pACYC-NheI_for and pACYC-NheI_rev. The reaction product was religated to **pACYC-NheI**. The plasmid pACYC-NheI was then enzymatically linearized using *Mlu*I and *Xho*I in order to ligate the *Mlu*I-*Xho*I fragment extracted from pCOLA_tacI/I fusing this enzymatic reaction to **pCK_0400**, the tacI/I promoter bearing version. Introducing the *araC*-P_{BAD} cassette into pCK_0400, thereby deleting *lacI-tacI* (one of the two *tacI* promoters), was done in a Gibson reaction with an *Ehe*I/*Eco*RI digested pCK_0400 vector part. The insert for this cloning step was obtained by a PCR using pCEP[19] template with pCEP_p15A_Gib_for and pCEP_p15A_Gib_rev as primer pair fusing the *Ehe*I/*Eco*RI linearized vector part with the insert to **pCK_0401**. Using the restriction enzymes *Nde*I and *Xho*I to open the vector pCK_0401 then

allowed the ligation of the *Nde*I/*Xho*I-*araE* bearing fragment extracted from pCK_0412 to be introduced into the vector, yielding **pCK_0402**. The *Nde*I/*Xho*I-*mtaA* part of pCK_0413 was introduced in the same manner into the above *Nde*I/*Xho*I opened vector pCK_0401 constructing **pCK_0403**.

The full length xefoampeptide *xfpS* was cloned into pCOLA_ara/tacI transcriptionally governed by P_{BAD}. To this end pCOLA_ara/tacI was amplified via PCR using the primer pair ck0002/ck0005 and fused in a Gibson cloning step with the PCR product of the *X. bovienii xfpS* having used ck0084/ck0092 as primers on *X. bovienii* gDNA as template resulting in **pCK_0564**.

The full length bearing *xfpS* vector pCK_0564 then was the template for the in frame fusion of the $^{C/N}$DD *paxBC* (*X. bovienii,* locus tags XBJ1_2152 and XBJ1_2151) creating two ribosomally coupled NRPS *xfpS* parts. The Gibson reaction consisted of the vector part in the form of *Hpa*I and *Asc*I linearized pCK_0564 and three PCR generated fragments. Two separate PCR reactions were performed on pCK_0564 as template employing either ck0148b with ck0290b and ck0216 with ck0289. The third fragment $^{C/N}$DD *paxBC* was amplified using *X. bovienii* gDNA in combination with the primer set ck0151 and ck0152c. The resulting plasmid was named **pCK_0584**.

Introducing docking domains between module one and two demanded the import of docking domains from a biosynthesis with a module junction E- to C-domain. For cloning pCK_0564 was subjected to a restriction enzyme digest using *Pst*I and *Acc*65I. The 2878 bp stretch within the XfpS coding region between *Pst*I and *Acc*65I was substituted in a four fragment (one vector, three inserts) Gibson cloning reaction reintroducing the *xfpS* coding parts and adding the *txlA*-^{C}DD to *txlB*-^{N}DD[20] (locus tags XBJ1_0774, XBJ1_0775, respectively). A PCR on pCK_0564 template using the primer set ck0285 with ck0085 and a separate PCR on the same template using ck0091 in combination with ck0284 resulted in two inserts for this cloning step. The third insert needed coding the $^{C/N}$DD was amplified from *X. bovienii* gDNA using the primer pair ck0251 and ck0252. The fusion of all four DNA fragments created **pCK_0597**. The cloning of a three-module three gene NRPS biosynthesis was achieved by ligation of the isolated *Pst*I/*Acc*65 restriction enzyme fragment of pCK_0597, to then ligate it into *Acc*65I/*Pst*I linearized pCK_0584 vector part, resulting in the plasmid **pCK_0620**.

Cloning of an in frame N-terminal Strep-tag II to *xfpS* was realised by a restriction enzyme digest of pCK_0564 with *Bst*EII and *Bgl*I in which the restriction enzyme site

linear ends lent themselves as homology arm entry site for two inserts being fused to the vector **pCK_0627**. The insert parts of the reaction introduced the Strep-tag II sequence. As template for the two insert PCRs pCK_0564 was taken in conjunction with the primer pair ck0348/ck0349 and in a separate PCR ck0350 in combination with ck0351. To attach an C-terminal Step-tag II to *xfpS,* pCK_0627 was linearized with *Sna*BI and *Sma*I. Two PCR generated fragments were then amplified taking pCk_0627 as template. The primer combination ck0327 and ck0328b lead to the first insert part while the primer set ck0326 used with ck0329b resulted in the second insert then all being fused in one Gibson cloning step to **pCK_0629**. The two times tagged *xfpS* plasmid version in combination with the $^{C/N}$DD located between module one and two was cloned using the *Acc*65I/*Pst*I DNA fragment from pCK_0597 to ligate it into the vector part of the *Acc*65I/*Pst*I sites of pCK_0629 cloning **pCK_0633**. The two times tagged *xfpS* plasmid version in combination with the $^{C/N}$DD located between module two and three was cloned using the *Hpa*I/*Asc*I DNA fragment from pCK_0584 to ligate it into the vector part of the *Hpa*I/*Asc*I sites of pCK_0629 cloning **pCK_0637**.

Deletion for DD-parts of the *txlA*-^{C}DD to *txlB*-^{N}DD introduced between the E1-domain and the C2-domain in pCK_0633 started in all three following cases with the *Acc*65I/*Pst*I linearized pCK_0633 as vector part. The two Inserts for the deletion of the *txlA*-^{C}DD were brought about in two separate PCRs using pCK0633 as template and ck0283/ck0285 and ck0284/ck0286 as primer pairs. The Gibson reaction of the three DNA fragments resulted in **pCK_0634**. The necessary inserts to delete the *txlB*-^{N}DD took pCK0633 as template for the PCR in combination with the primer pairs ck0283/0287 and in a second separate PCR ck0284/0288. The following Gibson cloning step yielded **pCK_0635**. The *txlA*-^{C}DD to *txlB*-^{N}DD double deletion, while preserving the translational coupling of the two coding regions, was achieved by performing two PCRs employing pCK_0634 as template using the primer pair ck0283/ck0285 and in a second PCR ck0284/ck0286. The resulting plasmid from the Gibson reaction of *Acc*65I/*Pst*I linearized pCK_0633 with the two pCK_0635 derived PCR fragments lead to **pCK_0641**.

Deletion of the ^{C}DD coded on pCK_0637 achieved by use of *Hpa*I and *Asc*I to linearize this vector for a Gibson cloning step. Two separate PCR inserts were amplified using the primer combination ck0289 with ck0293 and ck0290b with ck0294 and combined with the linearized vector to form **pCK0639**.

The vector **pCK_0714** was cloned by digesting pCK_0633 *Hpa*I and *Asc*I. In a PCR using ck0289/ck0290 and pCK_0637 as template the DNA fragment was amplified which was used in a Gibson cloning step to clone it into the *Hpa*I/*Asc*I linearized pCK-0633 leading to pCK_0714.

The primer pair ck0573/ck0574 was used with gDNA from *X. doucetiae* to amplify the *xabAB* DD coding region. This fragment was cloned in a Gibson reaction into the vector PCR fragment using ck0148b/0216 as primer pair and **pCK_0714** as template resulting in **pCK_0715**. The same vector PCR fragment was used to clone **pCK_0716** by fusing the amplified fragment from the *xacAB* NRPS coding region with the primer pair ck0575/ck0576 from *X. doucetiae* gDNA into the vector part in a Gibson reaction.

The PaxBC DD coding plasmid pCK_0714 was further changed by cloning two PCR products into the *Hpa*I/*Asc*I opened vector. The first PCR fragment using pCK0714 as template was amplified using ck0289 and ck0577 as primer while the second PCR used the same template but ck0290 and ck0578 as primer resulting in **pCK_0717**. Again the same *Hpa*I/*Asc*I digested pCK_0714 was used as the vector part in a Gibson reaction to clone two PCR fragments into it. The first PCR fragment was generated using pCK_0714 as template with the primer combination ck0289 and ck0579 and the second PCR fragment by use of ck0290 and ck0580 resulting in the vector **pCK_0718**.

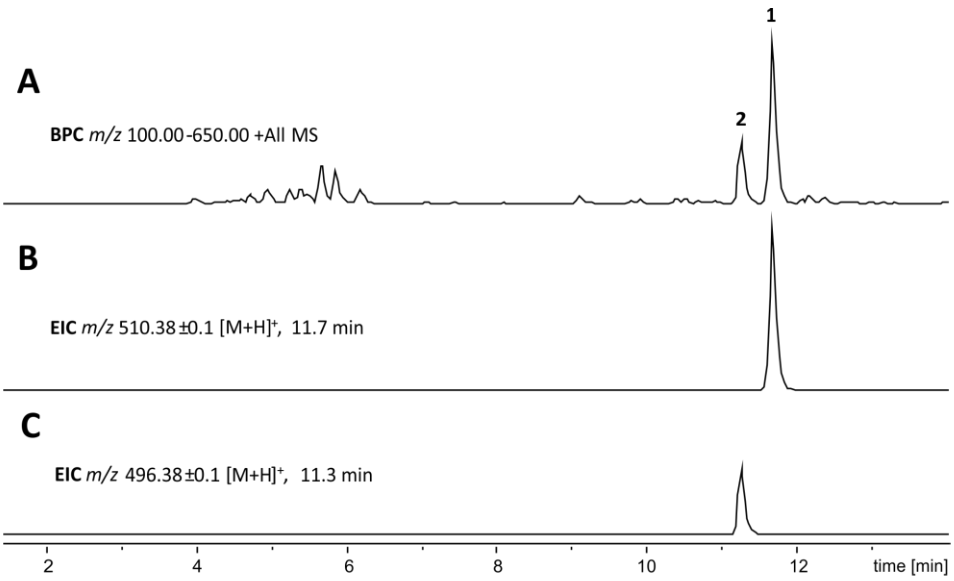

Figure S1. UPLC-ESI-MS analysis of XFP A/B (**1**/**2**) from *X. bovienii* heterologously produced in *E. coli*. **A** base peak chromatogram (BPC) of a methanolic extract of *E. coli* expressing the full length XfpS. **B** extracted ion chromatogram (EIC) of *m/z* 510.39 $[M+H]^+$, XFP A (**1**). **C** EIC of *m/z* 496.38 $[M+H]^+$, XFP B (**2**).

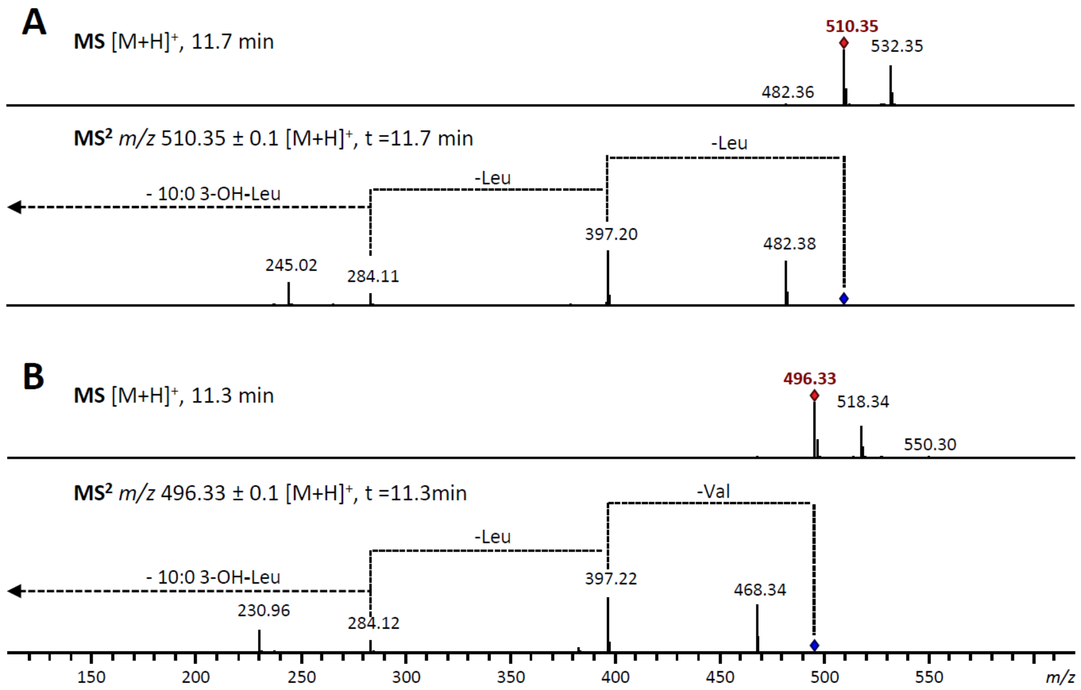

Figure S2. UPLC-ESI-MS, MS^2 analysis of XFP A/B (**1**/**2**) from *X. bovienii* heterologously produced in *E. coli*. **A** MS, MS^2 and neutral loss annotation analysis of XFP A (**1**). **B** MS, MS^2 and neutral loss annotation analysis of XFP B (**2**).

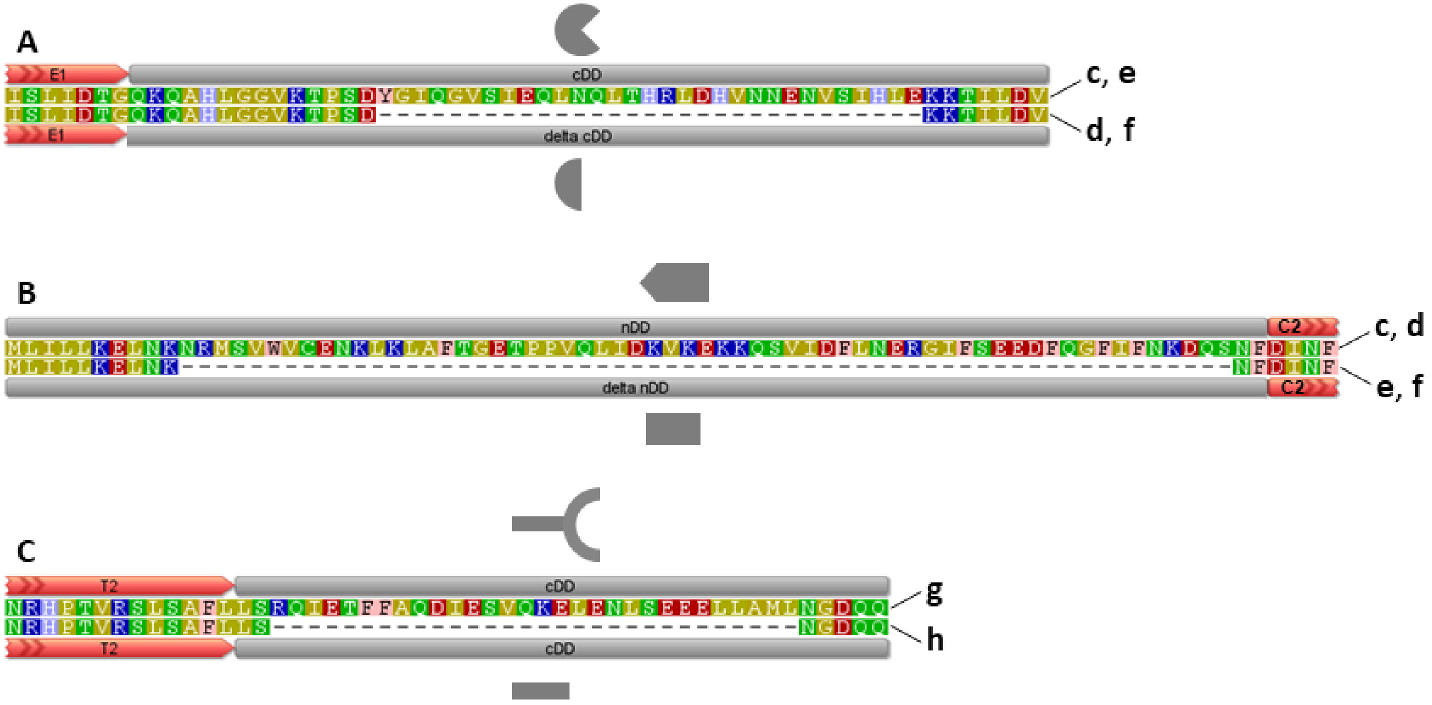

Fig S3. Alignment of DDs with ΔDD counterparts. **A** displays the E1 to C2 ^{C}DD alignment while in **B** the ^{N}DD subunit is aligned. **C** shows the alignment of the T2-^{C}DD. The relevant XfpS constructs carrying the displayed DD-version is indicated according to Fig. 2 and the DD symbols also correspond to Fig. 2.

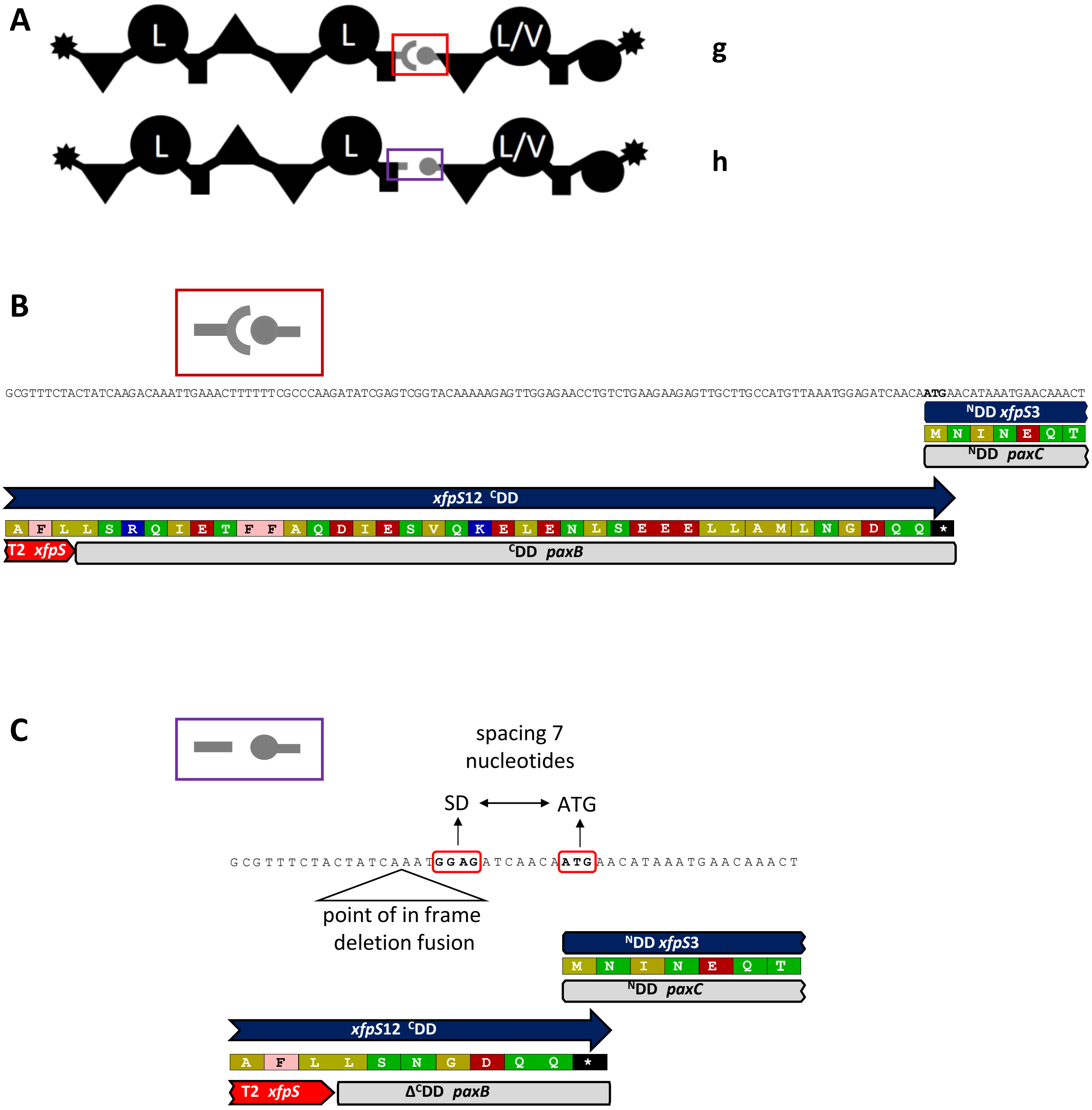

Figure S4. DD-deletion and translational coupling cloning approach. **A** schematic representation of the XfpS-12 and XfpS-3 constructs **g** and **h** (compare Fig. 2). The full length DD (in grey) regions are highlighted by a red rectangle while the deletion DD version is highlighted with a violet rectangle. **B** graphical representation of the DD coding region of the *xfpS12* to *xfpS3* (**g**) juxtaposed with the translational products (amino acids in one letter polarity colour code; blue bar and arrow: genes; grey bar: DD; red arrow: coding region for T-domain). **C** graphical representation of the DD coding region comprising the deleted area (SD: Shine-Dalgarno sequence; ATG indicating the start coding of XfpS-3).

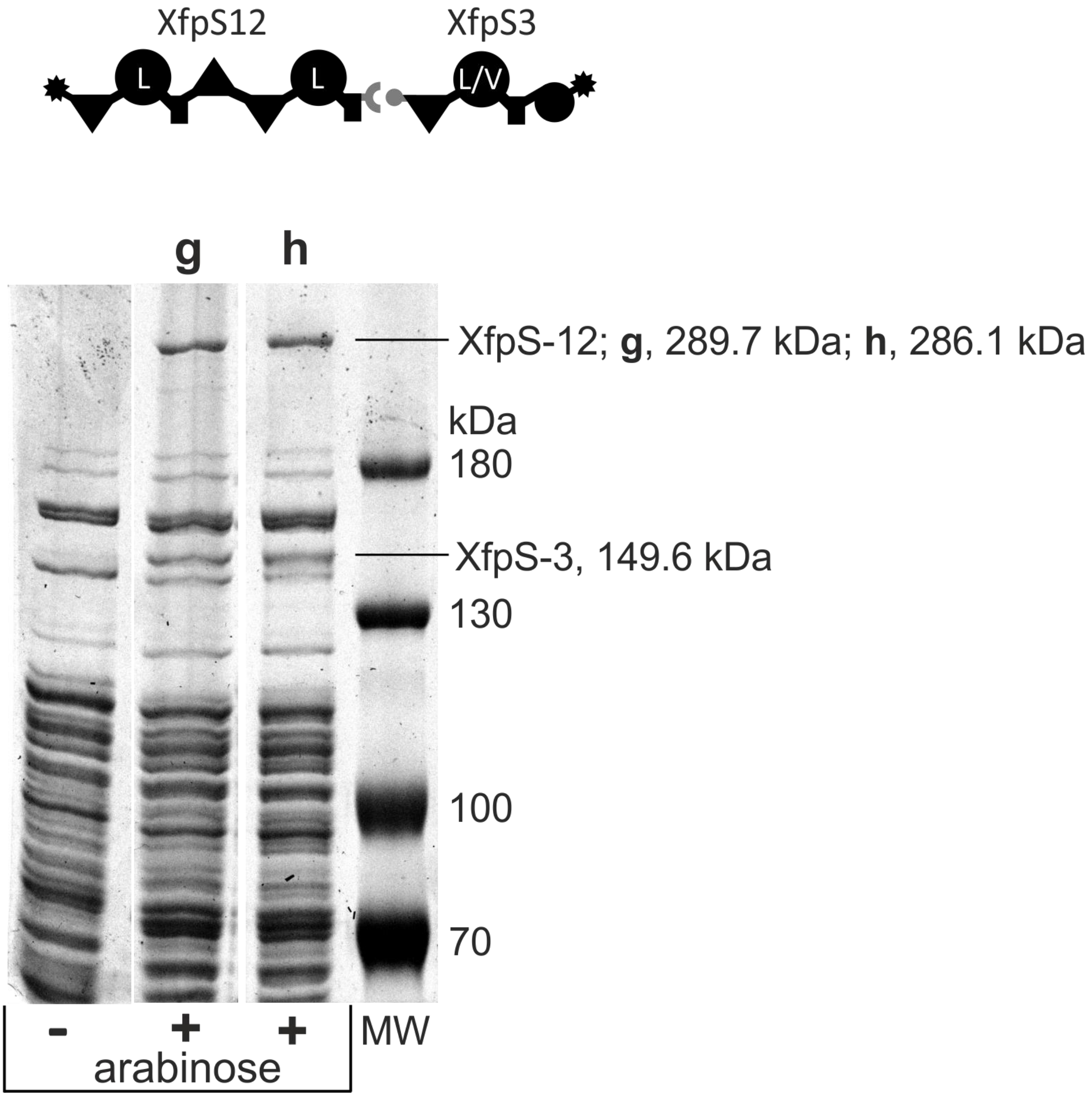

Figure S5. SDS-PAGE of soluble *E. coli* DH10B expressing XfpS constructs. XfpS constructs **g** and **h** (compare Fig. 2) were transcriptionally induced with arabinose, expressed at 22° for 24 h followed by extraction of soluble protein fraction as described above. The left lane represents a *xfpS* harbouring *E. coli* soluble protein extract being subjugated to the same temperature and expression profile with the exception of no arabinose being added. The emergence of the XfpS parts of **g** and **h** in response to arabinose induction are indicated

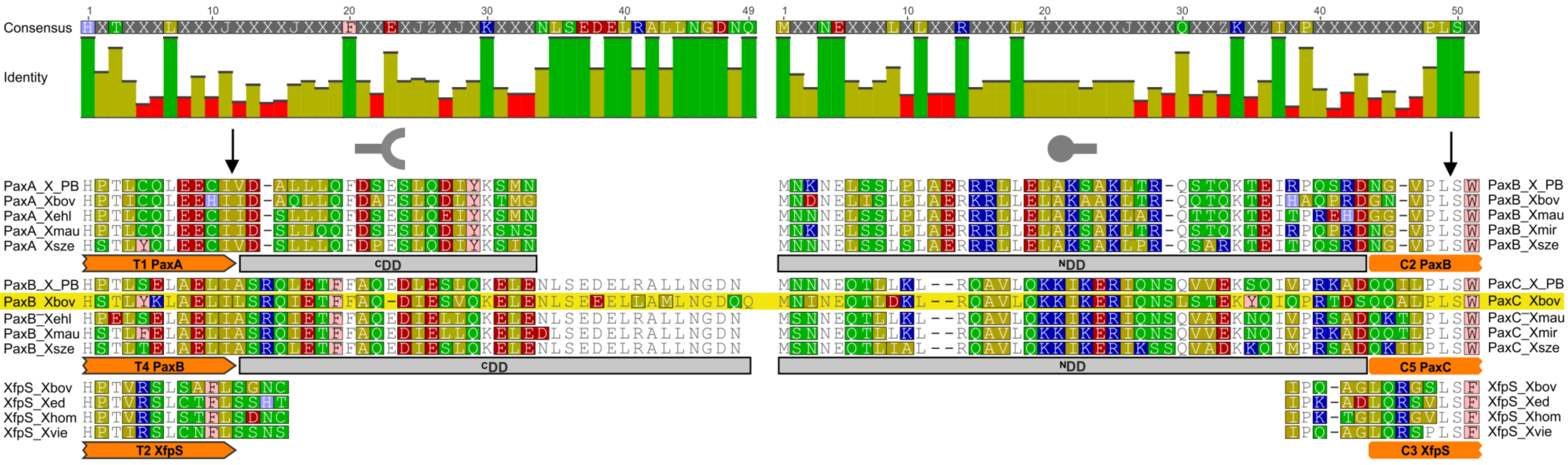

Figure S6. Alignment of NRPS ^{C}DD and ^{N}DD pairs of the PaxABC NRPS to XfpS T2 domain linker region. The consensus alignment is depicted above the alignment sequences with a ≥75% threshold implemented. Disagreements are highlighted in the alignment, T and C domains are represented as orange bars below the alignment subsections and ^{C}DD and ^{N}DDs parts are indicated as grey bars. The arrows above the alignment show the point of fusions for the DD insertions. The DD used in this work is highlighted in yellow.The abbreviation of the species are X_PB, *X.* PB62.4; Xbed, *X. beddingii, Xbov, X. bovienii;* Xehl, *X. ehlersii*; Xhom, *X. hominickii*; Xmau, *X. mauleonii*; Xsze, *X. szentirmaii*; Xvie, *X. vietnamensis*. Alignments were performed using the multiple alignment program MUSCLE (default parameters)[21].

a)

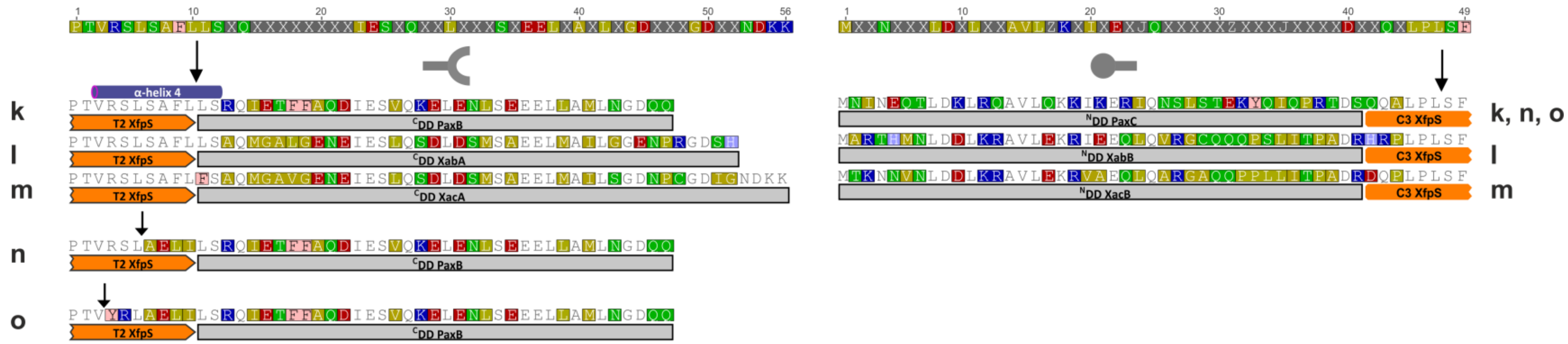

b)

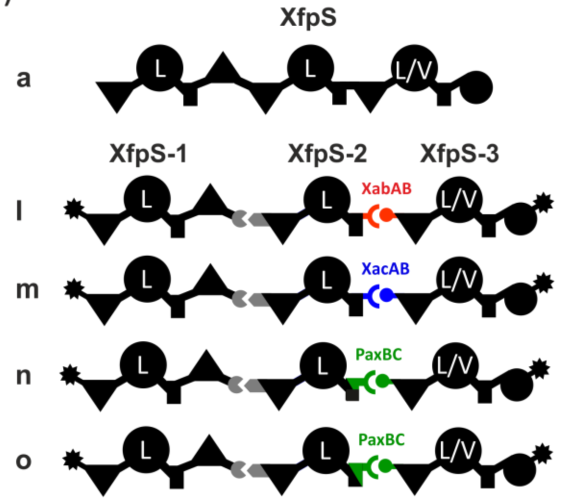

		production mg*l^{-1}; ± SD	% WT	ratio 1/2
a	1	1.21 ± 0.05	100	2.53
	2	0.48 ± 0.03	100	
l	1	0.08 ± 0.02	7	0.23
	2	0.35 ± 0.06	73	
m	1	0.12 ± 0.01	10	0.23
	2	0.52 ± 0.05	109	
n	1	-		
	2	-		
o	1	0.27 ± 0.02	22	0.50
	2	0.53 ± 0.05	111	

Figure S7. Alignment of NRPS ^{C}DD and ^{N}DD of constructs **k** to **o** and their respective XFP production data. The use of symbols is as in previous figures. In a) the α-helix4 at the end of the T-domain is indicated above the alignment (disagreements highlighted). The shifted point of fusion in **n** and **o** is indicated by an additional arrow. The different DD sets in b) are indicated in color and the corresponding production data is displayed as in Fig. 2.

Table S1. Primer used in this work.

primer	length (in bp)	Sequence 5' to 3'
pCOLA-NheI_for	21	AGGCATGCTAGACGCAGAAAC
pCOLA-NheI_rev	21	GTCCTAGAAGATGCCAGGAGG
tacI_dw	86	TATGCTAGCCTCCTGTGTGAAATTGTTATCCGCTCACAATTCC ACACATTATACGAGCCGATGATTAATTGTCAACAGCTCCTGCA
tacI_up	80	GGAGCTGTTGACAATTAATCATCGGCTCGTATAATGTGTGGAA TTGTGAGCGGATAACAATTTCACACAGGAGGCTAGCA
tacI_NcoI_up	88	CATGAGCTGTTGACAATTAATCATCGGCTCGTATAATGTGTGG AATTGTGAGCGGATAACAATTTCACACAGGAGGGAATTCCATGGC
tacI_NcoI_dw	88	AATTGCCATGGAATTCCCTCCTGTGTGAAATTGTTATCCGCTC ACAATTCCACACATTATACGAGCCGATGATTAATTGTCAACAGCT
pCEP_ColA_for	48	GATTCCCGACACCATCACTCTAGACAATTATGACAACTTGACGGCTAC
pCEP_ColA_rev	45	GAGCTCGAATTGCCATGGAATTCCTCCTGTTAGCCCAAAAAAACG
araE_Gib_for	49	AATTTCACACAGGAGGCTAGCATATGGTTACTATCAATACGGAATCTGC
araE_Gib_rev	43	GGTTTCTTTACCAGACTCGAGTCAGACGCCGATATTTCTCAAC
pACYC-NheI_for	28	GGAGTGTATACTGGCTTACTATGTTGGC
pACYC-NheI_rev	19	GGTGCTTTTGCCGTTACGC
pCEP_p15A_Gib_for	45	AGAGGCGGTTTGCGTATTGGGCAATTATGACAACTTGACGGCTAC
pCEP_p15A_Gib_rev	45	GAGCTCGAATTGCCATGGAATTCCTCCTGTTAGCCCAAAAAAACG
ck0005	18	CTGCAGGAGCTGTTGACA
ck0002	20	CATGGAATTCCTCCTGTTAG
ck0083	24	CATCACTGGAATCAGGCTTTTCTG
ck0084	45	TTTTGGGCTAACAGGAGGAATTCCATGGATAACATTCTGGCCTCG
ck0085	35	CCTGTTTTTGGCCCGTGTCGATAAGCGATATCAAG
ck0092	47	TGATTAATTGTCAACAGCTCCTGCAGCACAACAAATCAGCTCAATCC
ck0148b	20	TTTGCACAATTACGCCTCTG
ck0151	43	AAACCAGAGGCGTAATTGTGCAAAGGATAGCGGTAACGCCTGT
ck0152c	52	GTCAGATCTCTGAGCGCGTTTCTACTATCAAGACAAATTGAAACTTTTT TCG
ck0216	22	TAGAAACGCGCTCAGAGATCTG
ck0251	36	TGTTGGCCGGATATATCGCTTCAATTTTATTTCCAG
ck0252	36	ATATCGCTTATCGACACGGGCCAAAAACAGGCCCAC
ck0283	24	CATCACTGGAATCAGGCTTTTCTG
ck0284	24	TCTGTCAGTACCAACCTGATTTGG
ck0285	39	ATCCAAAATTGTTTTCTTATCACTGGGGGTTTTTACTCC
ck0286	35	CCCAGTGATAAGAAAACAATTTTGGATGTGTAACA
ck0287	57	GTAACAATATGTTGATATTACTGAAAGAACTAAATAAAAATTTTGATAT AAATTTTG
ck0288	46	GAAAGAACTAAATAAAAATTTTGATATAAATTTTGTTGATCCCAGC
ck0289	22	CGACTGAAAGCAGTGTCTGCTG
ck0290b	24	GTCAACAAAACCGCATGTAAAGTC
ck0293	39	TCTGAGCGCGTTTCTACTATCAAATGGAGATCAACAATG
ck0294	41	CGTTTCTACTATCAAATGGAGATCAACAATGAACATAAATG
ck0326	22	TGATGATGCATGGTTACTCACC
ck0327	21	TGGCCATTGGCTACTATCGTG
ck0328b	47	AACTGTGGATGACTCCATCCTACGCTAGCATCCACCAGCTCCAACAC
ck0329b	48	AGGATGGAGTCATCCACAGTTCGAGAAGTAATTAACCTAGGCTGCTGC
ck0331	21	GGATTAGTTGCTGCCACTTCC
ck0348	26	CACTGCGTCTTTTACTGGCTCTTCTC

ck0349	57	AGCAGAAGCTTTTTCAAATTGAGGGTGAGACCAACCCATGGAATTC CTCCTGTTAGC
ck0350	53	TCTCACCCTCAATTTGAAAAAGCTTCTGCTTCCATGGATAACATTC TGGCCTC
ck0351	19	CAGATAAGCCGCTCCCGCT
ck0573	43	GTCAGATCTCTGAGCGCGTTTCTATTATCAGCGCAAATGGGAG
ck0574	41	AAACCAGAGGCGTAATTGTGCAAAAGAGAGCGGCAGAGGCC
ck0575	44	GTCAGATCTCTGAGCGCGTTTCTATTTTCAGCTCAAATGGGTGC
ck0576	41	AAACCAGAGGCGTAATTGTGCAAAAGAGAGCGGCAGAGGCT
ck0577	44	GTATAAGTTCTGCCAGAGATCTGACGGTAGGATGCCGGTTTAAC
ck0578	50	CAGATCTCTGGCAGAACTTATACTATCAAGACAAATTGAAACTTTTTTC G
ck0579	44	GTATAAGTTCTGCCAGACGGTAGACGGTAGGATGCCGGTTTAAC
ck0580	50	CTACCGTCTGGCAGAACTTATACTATCAAGACAAATTGAAACTTTTTTC G

Table S2. Plasmids used in this work.

Plasmid	Size (in bp)	Description, genotype	Ref
pCOLADuet	3719	ori ColA, kan^R, twice the T7lac promoter, *lacI*	[22]
pACYCDuet	4008	ori p15A, cam^R, twice the T7lac promoter, *lacI*	[22]
pCOLA_ tacI/I	3682	ori ColA, kan^R, twice the *tacI* promoter, *lacI*	this work
pCOLA_ara/ tacI	3345	ori ColA, kan^R, *araC-P_{BAD}* and *tac*I	[17]
pCK_0412	4710	ori ColA, kan^R, *araC-P_{BAD}* and *tac*I-*araE*	this work
pCK_0413	4143	ori ColA, kan^R, *araC-P_{BAD}* and *tac*I-*mtaA*	this work
pACYC-Nhe	3989	ori p15A, cam^R, twice T7 promoter, *lac*I, NheI site abrogated	this work
pCK_0400	3951	ori p15A, cam^R, *lac*I, twice *tac*I	this work
pCK_0402	5256	ori p15A, cam^R, *araC-P_{BAD}* and *tac*I-*araE*	this work
pCK_0403	4689	ori p15A, kan^R, *araC-P_{BAD}* and *tac*I-*mtaA*	this work
pCK_0564	14498	pCOLA_ara/tacI, *P_{BAD} xfpS*, **a**	this work
pCK_0584	14689	pCOLA_ara/tacI, *P_{BAD}*-two gene *xfpS-1* and *xfpS-23* translationally coupled, T2-**^{C}DD ^{N}DD**-C3 from *X. bovienii paxBC*	this work
pCK_0597	14838	pCOLA_ara/tacI, *P_{BAD}*-two gene *xfpS-1* and *xfpS-23* translationally coupled, E1-**^{C}DD ^{N}DD**-C2 from *X. bovienii txlAB*	this work
pCK_0620	15029	pCOLA_ara/tacI, *P_{BAD}*-three gene gene *xfpS-1, xfpS-2* and *xfpS-3*, $^{C/N}$DD-*txlAB* inserted between module one and two, $^{C/N}$DD-*paxBC* inserted between module two and three, **i**	this work
pCK_0627	14540	pCOLA_ara/tacI, *P_{BAD} xfpS-123* with N-terminal Strep-tag®II	this work
pCK_0629	14552	pCOLA_ara/tacI, *P_{BAD} xfpS-123* with N- and C-terminal Strep-tag®II, **b**	this work
pCK_0633	14892	pCOLA_ara/tacI, *P_{BAD}* two gene *xfpS-1* and *xfpS-23* translationally coupled with E1-^{C}DD ^{N}DD-C2 from *X. bovienii txlAB*, N-/C-terminal Strep-tag®II, **c**	this work
pCK_0634	14799	pCOLA_ara/tacI, *P_{BAD}* two gene *xfpS-1* and *xfpS-23* translationally coupled with E1-^{C}DD ^{N}DD-C2 from *X. bovienii txlAB,* 31 ^{C}DD-codons deleted; N-/C-terminal Strep-tag®II, **d**	this work
pCK_0635	14709	pCOLA_ara/tacI, *P_{BAD}* two gene *xfpS-1* and *xfpS-23* translationally coupled with E1-^{C}DD ^{N}DD-C2 from *X. bovienii txlAB,* 61 ^{N}DD-codons deleted; N-/C-terminal Strep-tag®II, **e**	this work

pCK_0637	14743	pCOLA_ara/tacI, P_{BAD}-two gene *xfpS-12* and *xfpS-3* translationally coupled, T2-^{C}**DD** ^{N}**DD**-C3 from *X. bovienii paxBC*; N-/C-terminal Strep-tag®II, **g**	this work
pCK_0639	14653	pCOLA_ara/tacI, P_{BAD}-two gene *xfpS-12* and *xfpS-3* translationally coupled, T2-^{C}**DD** ^{N}**DD**-C3 from *X. bovienii paxBC,* 30 ^{C}DD-codons deleted; N-/C-terminal Strep-tag®II, **h**	this work
pCK_0641	14616	pCOLA_ara/tacI, P_{BAD} two gene *xfpS-12* and *xfpS-3* translationally coupled with E1-^{C}DD ^{N}DD-C2 from *X. bovienii txlAB,* 31 ^{C}DD-codons deleted and 61 ^{N}DD-codons deleted, N-/C-terminal Strep-tag®II, **f**	this work
pCK_0714	15083	pCOLA_ara/tacI, P_{BAD}-three gene gene *xfpS-1, xfpS-2* and *xfpS-3*, $^{C/N}$DD-*txlAB* inserted between module one and two, $^{C/N}$DD-*paxBC* inserted between module two and three; N-/C-terminal Strep-tag®II, **k**	this work
pCK_0715	15101	pCOLA_ara/tacI, P_{BAD}-three gene gene *xfpS-1, xfpS-2* and *xfpS-3*, $^{C/N}$DD-*txlAB* inserted between module one and two, $^{C/N}$DD-*xabAB* inserted between module two and three; N-/C-terminal Strep-tag®II, **l**	this work
pCK_0716	15100	pCOLA_ara/tacI, P_{BAD}-three gene gene *xfpS-1, xfpS-2* and *xfpS-3*, $^{C/N}$DD-*txlAB* inserted between module one and two, $^{C/N}$DD-*xacAB* inserted between module two and three; N-/C-terminal Strep-tag®II, **m**	this work
pCK_0717	15083	pCOLA_ara/tacI, P_{BAD}-three gene gene *xfpS-1, xfpS-2* and *xfpS-3*, $^{C/N}$DD-*txlAB* inserted between module one and two, $^{C/N}$DD-*paxBC* inserted between module two and three; T4 α-helix4 PaxB AELI inserted into T2 XfpS N-/C-terminal Strep-tag®II, **n**	this work
pCK_0718	15083	pCOLA_ara/tacI, P_{BAD}-three gene gene *xfpS-1, xfpS-2* and *xfpS-3*, $^{C/N}$DD-*txlAB* inserted between module one and two, $^{C/N}$DD-*paxBC* inserted between module two and three; N-/C-terminal Strep-tag®II, **o**	this work

Table S3. Bacterial strains used in this work

Strain	Genotype	ref
E.coli DH10B::*mtaA*	K-12 F– mcrA Δ(mrr-hsdRMS-mcrBC) ϕ80lacZΔM15 ΔlacX74 recA1 endA1 araD139 Δ(ara-leu)7697 galU galK λ– rpsL(StrR) nupG endD::mtaA	[23]
Xenorhabdus bovienii SS-2004	wild type	[24]

References

[1] E. Dehling, G. Volkmann, J. C. J. Matern, W. Dörner, J. Alfermann, J. Diecker, H. D. Mootz, *J. Mol. Biol.* **2016**, *428*, 4345.

[2] C. Hacker, X. Cai, C. Kegler, L. Zhao, A. K. Weickhmann, J. P. Wurm, H. B. Bode, J. Wöhnert, *Nat. Commun.* **2018**, *9*, 4366.

[3] C. D. Richter, D. Nietlispach, R. W. Broadhurst, K. J. Weissman, *Nat. Chem. Biol.* **2008**, *4*, 75.

[4] D. P. Dowling, Y. Kung, A. K. Croft, K. Taghizadeh, W. L. Kelly, C. T. Walsh, C. L. Drennan, *Proc. Natl. Acad. Sci. U. S. A.* **2016**, *113*, 12432.

[5] S. Kosol, A. Gallo, D. Griffiths, T. R. Valentic, J. Masschelein, M. Jenner, Santos, Emmanuel L. C. de los, L. Manzi, P. K. Sydor, D. Rea et al., *Nat. Chem.* **2019**, *11*, 913.

[6] S. A. Samel, G. Schoenafinger, T. A. Knappe, M. A. Marahiel, L.-O. Essen, *Structure* **2007**, *15*, 781.

[7] M. S. Klausen, M. C. Jespersen, H. Nielsen, K. K. Jensen, V. I. Jurtz, C. K. Sønderby, M. O. A. Sommer, O. Winther, M. Nielsen, B. Petersen et al., *Proteins: Struct., Funct., Bioinf.* **2019**, *87*, 520.

[8] A. Tanovic, S. A. Samel, L.-O. Essen, M. A. Marahiel, *Science* **2008**, *321*, 659.

[9] X. Cai, S. Nowak, F. Wesche, I. Bischoff, M. Kaiser, R. Fürst, H. B. Bode, *Nat. Chem.* **2017**, *9*, 379.

[10] Q. Zhou, F. Grundmann, M. Kaiser, M. Schiell, S. Gaudriault, A. Batzer, M. Kurz, H. B. Bode, *Chem.: Eur. J.* **2013**, *19*, 16772.

[11] S. W. Fuchs, A. Proschak, T. W. Jaskolla, M. Karas, H. B. Bode, *Org. Biomol. Chem.* **2011**, *9*, 3130.

[12] X. Cai, L. Zhao, H. B. Bode, *Org. Lett.* **2019**, *21*, 2116.

[13] J. Watzel, C. Hacker, E. Duchardt-Ferner, H. Bode, J. Wöhnert, *ACS Chem. Biol.* **2020**, accepted.

[14] a) X. Zhang, T. Reeder, R. Schleif, *Journal of molecular biology* **1996**, *258*, 14; b) R. Schleif, FEMS Microbiol. Rev. **2010**, *34*, 779.

[15] I. Orhan, B. Sener, M. Kaiser, R. Brun, D. Tasdemir, *Mar. Drugs* **2010**, *8*, 47.

[16] S. Nwaka, A. Hudson, Nat. Rev. Drug Discovery **2006**, *5*, 941.

[17] W. Lorenzen, T. Ahrendt, K. A. J. Bozhüyük, H. B. Bode, *Nat. Chem. Biol.* **2014**, *10*, 425.

[18] D. G. Gibson, L. Young, R.-Y. Chuang, J. C. Venter, C. A. Hutchison, H. O. Smith, *Nat. Methods* **2009**, *6*, 343.

[19] E. Bode, A. O. Brachmann, C. Kegler, R. Simsek, C. Dauth, Q. Zhou, M. Kaiser, P. Klemmt, H. B. Bode, *ChemBioChem* **2015**, *16*, 1115.

[20] M. Kronenwerth, K. A. J. Bozhüyük, A. S. Kahnt, D. Steinhilber, S. Gaudriault, M. Kaiser, H. B. Bode, *Chem.: Eur. J.* **2014**, *20*, 17478.

[21] a) R. C. Edgar, *Nucleic Acids Res.* **2004**, *32*, 1792; b) R. C. Edgar, S. Batzoglou, *Curr. Opin. Struct. Biol.* **2006**, *16*, 368.

[22] Merck_Novagen.

[23] O. Schimming, F. Fleischhacker, F. I. Nollmann, H. B. Bode, *ChemBioChem* **2014**, *15*, 1290.

[24] J. M. Chaston, G. Suen, S. L. Tucker, A. W. Andersen, A. Bhasin, E. Bode, H. B. Bode, A. O. Brachmann, C. E. Cowles, K. N. Cowles et al., *PloS one* **2011**, *6*, e27909.
